# Screening attendance disparities among Hungarian-speaking Roma and non-Roma women in central and eastern European countries

**DOI:** 10.3389/fpubh.2023.1292598

**Published:** 2023-12-22

**Authors:** Noémi Mózes, Johanna Takács, Zoltan Ungvari, Helga Judit Feith

**Affiliations:** ^1^Department of Public Health, Faculty of Medicine, Semmelweis University, Budapest, Hungary; ^2^Doctoral School of Health Sciences, Semmelweis University, Budapest, Hungary; ^3^Department of Social Sciences, Faculty of Health Sciences, Semmelweis University, Budapest, Hungary; ^4^Vascular Cognitive Impairment, Neurodegeneration, and Healthy Brain Aging Program, Department of Neurosurgery, University of Oklahoma Health Sciences Center, Oklahoma City, OK, United States; ^5^Oklahoma Center for Geroscience and Healthy Brain Aging, University of Oklahoma Health Sciences Center, Oklahoma City, OK, United States; ^6^International Training Program in Geroscience, Doctoral School of Basic and Translational Medicine/Department of Public Health, Semmelweis University, Budapest, Hungary; ^7^Department of Health Promotion Sciences, College of Public Health, University of Oklahoma Health Sciences Center, Oklahoma City, OK, United States

**Keywords:** minority, Roma women, screening tests attendance, health insurance, access to healthcare

## Abstract

The Roma populations in Central and Eastern Europe are two to three times more likely to have unmet health needs compared to non-Roma residents. The aim of the present study was to investigate the disparity in screening attendance between Hungarian-speaking Roma (R) and non-Roma (nR) women in Hungary (HU-R:322; nR:294), Romania (RO-R:258; nR:183), and Slovakia (SK-R:146; nR:163), while also identifying the factors that influence attendance at any kind of screening tests in both populations. In order to examine these factors, a multiple binary logistic regression was conducted. The findings revealed significant associations between attendance at any kind of screening tests and certain factors among different groups. Among Hungarian Roma women, it was found that having a chronic disease and smoking were linked to attendance at any kind of screening tests (*p* = 0.009). Specifically, having a chronic disease increased the odds of attendance (OR = 1.71 [1.01, 2.90]), while smoking decreased the odds (OR = 0.57[0.365, 0.91]). In Romania, the study found that not having health insurance decreased the odds of attendance among Roma women (OR = 0.50 [0.27, 0.91]), whereas having a chronic disease increased the odds (OR = 2.87 [1.44, 5.72]) (*p* = 0.006). Among non-Roma women in Romania, physical inactivity was associated with a decreased likelihood of attendance at any kind of screening tests (OR = 0.48 [0.25, 0.95]). Among Slovakian Roma women, not having health insurance (OR = 0.09[0.02, 0.36]) and smoking (OR = 0.25[0.11, 0.61]) were found to decrease the odds of attendance (*p* < 0.001). On the other hand, non-Roma women in Slovakia with chronic diseases were more likely to attend at any kind of screening tests (OR = 2.52[1.12, 5.66]). Our research emphasizes the impact of lacking health insurance on screening attendance, particularly among the Roma population. It also highlights the significance of health-related behaviours such as smoking and physical inactivity in relation to missed screening tests, which in turn contribute to the development of non-communicable diseases. Therefore, promoting targeted screening programs for the Roma community is crucial to ensure their access to screening tests, especially in cases of chronic illnesses.

## 1 Introduction

According to the European Commission, the estimated number of the Roma population is 10–12 million (1), with 50%–60% of them living in Central and Eastern Europe (CEE). The European Union has highlighted the higher burden of disease among the Roma people (2–5); despite the fact Roma people still have an increased disparity accessing healthcare when compared to other populations (6–12).

The Roma population in Central and Eastern Europe faces numerous challenges when it comes to accessing healthcare, resulting in a higher likelihood of unmet health needs compared to their non-Roma counterparts (13, 14). Multiple studies have identified various barriers, including poverty, lower education levels, limited health literacy, administrative obstacles related to displacement and lack of identification documents (15, 16). Cultural and linguistic differences, coupled with discriminatory behaviour from healthcare staff, further contribute to this concerning situation. Additionally, the Roma population’s lack of trust in the healthcare system poses significant challenges. The lower educational attainment among Roma individuals is a crucial factor contributing to their poorer health outcomes and limited knowledge of preventive measures (17–23). Addressing these issues requires a multifaceted approach. Firstly, it is imperative to provide comprehensive education to healthcare workers regarding Roma culture, enabling them to better understand and address the unique needs of this community. Additionally, initiatives should be implemented to encourage Roma children to pursue careers in health professions, fostering representation and cultural competence within the healthcare workforce (24, 25).

Access to healthcare poses significant challenges for the Roma population in Central and Eastern Europe (CEE), particularly regarding health insurance coverage (26). While comprehensive antenatal care is provided to all women in Hungary, Romania, and Slovakia, there are differences in the initial check-up process. In Romania, where the first check-up is tied to cost-reimbursement, financially constrained women tend to postpone visits (27–29). Roma women often experience inadequate healthcare contact, even when they possess health insurance, particularly in cases of chronic illnesses (30). Although Roma individuals generally understand the role of lifestyle factors in reducing cancer risk, there is limited confidence in the effectiveness of preventive measures, as shared traditional cultural beliefs do not always influence individual behaviour (31).

In Romania, socioeconomic status has a greater impact on access to healthcare compared to the EU average, and Roma women face higher unmet health needs due to financial constraints (32). Discriminatory practices, such as avoiding physical contact with Roma patients or diagnosing remotely without proper examination, increase the risk of misdiagnosis. Lengthy wait times and insufficient explanation of examination results further compound the problem. To address these issues, health mediators have been trained in Slovakia and Romania to facilitate doctor-patient communication and enhance Roma individuals’ health knowledge and awareness (33–35). Difficulties in seeking care include financial barriers, lack of health insurance, employment status, limited-service availability, waiting times, communication barriers, cultural differences, distrust, fear, and anxiety (36). In Hungary, a patient’s representative system has been implemented to protect patients’ rights and provide assistance in understanding and asserting those rights (37).

In Slovakia, access to health insurance is similar among the Roma and non-Roma populations (7, 25). However, Roma individuals are more likely to report difficulties in accessing healthcare, particularly women with lower education levels. Perceived social support reduces the likelihood of reporting healthcare inequalities for both Roma and non-Roma individuals (38). Healthcare inequalities are driven by Antigypsyism, exclusion, and the fact that many Roma live in settlements lacking basic infrastructure and services (39).

In Hungary, both Roma and non-Roma populations have almost complete health insurance coverage. However, over half of Roma individuals seldom or never utilize health services, including general practitioners (7). Barriers to screening among Hungarian Roma women include concerns about being exposed during the screening tests, fear of abnormal results, and the belief that screening procedures are painful (40). Additionally, lack of knowledge and cultural differences hinder effective cooperation between healthcare providers and Roma patients (37, 41).

The COVID-19 pandemic has further highlighted the challenges faced by vulnerable groups, including the Roma minority, in accessing healthcare and receiving routine care for chronic conditions (42–45). The long-term consequences of reduced screening tests rates, particularly among marginalized communities like the Roma, will place an additional burden on society. Therefore, it is crucial to focus on the health needs of national minorities, especially Hungarian-speaking Roma women in Romania and Slovakia. As women, they play significant roles in maintaining family health and serve as important role models (26, 32, 46, 47).

Given the difficulties in accessing this small population group, especially during the COVID-19 epidemic, it was important to conduct research that gathered information from as many women belonging to this minority as possible. The present study aimed to examine the disparities in screening attendance between Hungarian-speaking Roma and non-Roma women in Hungary, Romania, and Slovakia (CEEc). We explored the attitudes of Roma women toward healthcare in these three countries, as well as the reasons for their lack of attendance at any kind of screening tests. Furthermore, we investigated factors influencing the attendance at any kind of screening tests among both Roma and non-Roma women. Our research is unique in that it focuses on women, who play crucial roles in family health, and employs a consistent methodology across CEEc among Hungarian-speaking Roma and non-Roma populations.

In the study, we focused on “any kind of screening tests.” By “any kind of screening tests,” we mean screening tests carried out for organised public health purposes (cervical cancer, breast cancer, colorectal cancer), as well as screening tests in primary care (e.g., blood pressure, blood glucose measurement) and specialist care (e.g., skin, vision examination).

## 2 Materials and methods

### 2.1 Participants and data collection

Our cross-sectional research was conducted in Central and Eastern European countries (CEEc) from September 2020 to March 2022, with the majority of surveys carried out in 2021. The study spanned 19 months due to several factors. Firstly, reaching and involving the Roma population in rural areas is challenging for any scientific research. Secondly, conducting research in three countries posed significant recruitment difficulties. Lastly, the COVID-19 pandemic further impacted the timeline of the study (48, 49). It is important to note that, based on available data in all three countries, there were no long-term suspensions of screening tests during our research period starting from September 2020 (50–52).

To obtain a representative sample, the Hungarian-speaking Roma regions were divided into eight regions, with the aim of achieving nearly equal proportions of participants from each region (five regions in Hungary, two regions in Romania, and one region in Slovakia). The study was conducted in 21 rural municipalities in Hungary, 15 in Romania, and 6 in Slovakia, excluding capital cities. Our target group comprised self-reported Roma and non-Roma residents. We specifically focused on municipalities where Roma and non-Roma individuals lived together. The number of non-Roma participants in each region corresponded to the number of Roma individuals interviewed in the same region. The Slovak and Romanian samples were obtained from historical Hungarian territories, from individuals who identified themselves as Hungarian-speaking Roma or Hungarian-speaking non-Roma. To successfully recruit the Roma population, we collaborated with organizations that had strong connections with the minority, such as municipal settlements, Roma municipalities, Family Care Centres, Non-governmental Organizations, the Maltese Charity Service, the Catholic Charity, and the Reformed Church. These organizations assisted us in reaching the target population.

**Figure 1:**
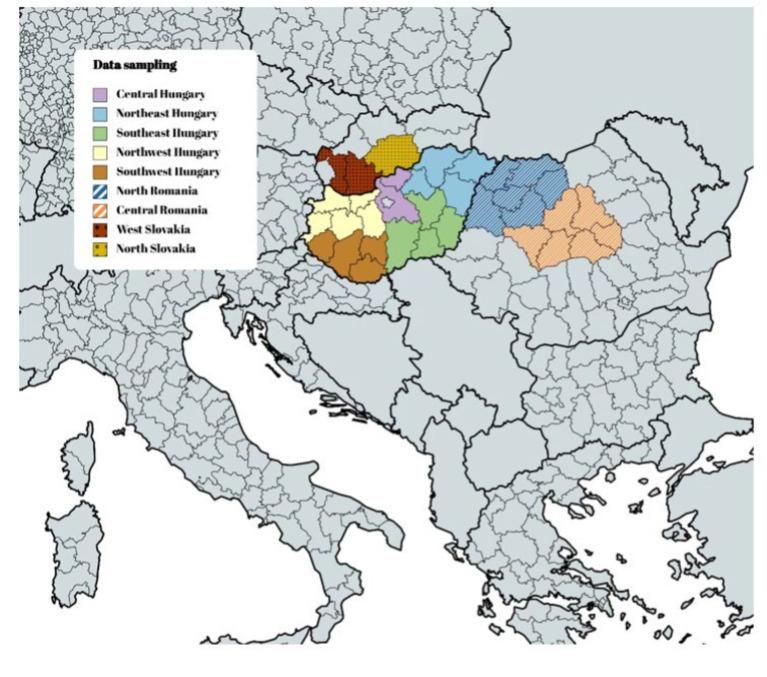
The research sample by geographical region. Source: Own editing based on the MapChart Protram (https://www.mapchart.net/).

Sample size calculation before the data collection was complicated since it is difficult to estimate the number of Roma populations in Central and Eastern Europe. Based on the convenience sampling we have selected participants from all geographical regions of Hungary. When designing the survey, the aim was to approach 50% of the Roma population in Hungary, after dividing the Roma population in Hungary into five regions: Northeast, Southeast, Northwest, Southwest, and Central Hungary. In the other half of the Hungarian-speaking Roma sample, the number of Roma participants from Romania and Slovakia should be two-thirds to one-third (given that there are two regions with Hungarian-speaking Roma in Romania and two regions with Hungarian-speaking Roma in Slovakia). Finally, due to the aggravating circumstances, Roma in Hungary are 44% of the sample, Roma in Romania 35%, and Roma in Slovakia 20%. The data sampling is illustrated in Figure 1.

The inclusion criteria for the sample were being over 18 years of age, able to speak Hungarian, and self-identifying as Roma in the Roma sample and non-Roma in the non-Roma sample, and completion of the questionnaire. Exclusion criteria included being under 18 years of age, lacking competency, and refusing to complete the questionnaire in its entirety. However, the exclusion criterion did not apply to individuals who were unable to read or write, as interviewers were present to assist them in completing the questionnaire. Detailed information about the study and its procedures was provided to all participants. For those participants who were unable to read and write have been informed orally. The consent form and the questionnaire were also read out loud. The respondent verbally expressed his/her agreement and consent. The fact of the informing was recorded on the consent form, which the respondent confirmed by completing the questionnaire. Our research received approval from the Scientific and Research Ethics Committee (decision IV/5210-2/2020/EKU).

The questionnaire was available in both online and paper formats, and participants could choose their preferred mode of completion. There were no differences between the online and paper versions of the questionnaire. In all cases, completion took place at the research site, and participants who completed the questionnaire online had the opportunity to ask questions of the interviewer. In the online questionnaire, respondents could only proceed to the next question once the previous question was answered. In the database, online completion accounted for 39.6% of responses, while paper completion accounted for 60.4%. For online completion, the questionnaire included branched sections for specific Roma-related questions. Trained interviewers were also available to assist non-Roma participants as needed. Questionnaires were administered to Roma respondents in community centres by social workers or trained interviewers, providing assistance in cases of limited literacy or illiteracy.

This study was performed in line with the principles of the Declaration of Helsinki. Ethical clearance: ETT TUKEB IV/3495-4/ 2021/EKU.

### 2.2 Measures

Our self-compiled questionnaire collected information on the following aspects:

1. Socioeconomic characteristics (refer to Table 1).
2. Attendance at any kind of screening tests in the year before the survey (referred to as ASBS), indicating whether participants participated in any kind of screening tests.
3. Health insurance coverage and presence of chronic diseases.
4. Health status is categorized as feeling healthy or ill.
5. Health behaviours, including smoking status and physical activity level. Participants were classified as active if they engaged in daily or regular exercise.

### 2.3 Data analysis

Descriptive statistics and relative frequency distributions were employed to describe the sample, presenting data as mean ± standard deviation (SD) and proportions. To compare differences between the Roma and non-Roma samples, a robust independent samples t-test was used, with effect size measured using Hedges’s g. Cross-tabulation, Pearson’s chi-square test, and Fisher’s exact test were used to examine associations between ethnicity and the studied variables, with effect sizes measured using Phi or Cramer’s V. Lastly, multiple binary logistic regression was performed to explore associations between ASBS and the studied variables. Separate models were constructed for each country by ethnicity, with the calculation of adjusted odd ratios. The significance level was set at 0.05. Statistical analysis was conducted using IBM SPSS Statistics for Windows, Version 25.0 (IBM Corp. Released 2017, Armonk, NY, United States: IBM Corp.).

## 3 Results

### 3.1 Study sample

The study included 1,366 female individuals from three countries: Hungary, Romania, and Slovakia (Hungary-Roma:322; non-Roma:294, Romania-Roma:258; non-Roma:183, Slovakia-Roma:146; non-Roma:163). The socio-economic characteristics of the sample, including both Roma and non-Roma women from the three countries, are presented in Table 1 (Supplementary File S1).

**Table 1:** Socio-economic characteristics among Roma and non-Roma women in Hungary, Romania, and Slovakia.

	Hungary (*n* = 616)			Romania (*n* = 441)			Slovakia (*n* = 309)		
	Roma (*n* = 322)	non-Roma (*n* = 294)	*p*[ES]	Roma (*n* = 258)	non-Roma (*n* = 183)	*p*[ES]	Roma (*n* = 146)	non-Roma (*n* = 163)	*p*[ES]
Age, M ± SD	44.70 ± 13.99	46.29 ± 15.07	0.174[0.11]	39.37 ± 14.25	40.6 ± 16.64	0.406[0.08]	39.32 ± 15.0	39.67 ± 14.19	0.833[0.02]
Age groups, *n*(%)									
18–34	83(25.8)_a_	74(25.2)_a_		105(40.7)_a_	75(41.0)_a_		60(41.1)_a_	64(39.2)_a_	
35–44	73(23.0)_a_	54(18.4)_a_	0.222	62(24.0)_a_	35(19.1)_a_	0.259	32(21.9)_a_	46(28.2)_a_	0.423
45–64	141(43.7)_a_	132(44.8)_a_	[0.08]	78(30.3)_a_	56(30.6)_a_	[0.09]	46(31.5)_a_	41(25.2)_a_	[0.09]
65+	24(7.5)_a_	34(11.6)_a_		13(5.0)_a_	17(9.3)_a_		8(5.5)_a_	12(7.4)_a_	
Education, *n*(%)									
Primary school	194(60.2)_a_	44(15.0)_b_	<0.001 [0.56]	210(81.4)_a_	53(29.0)_b_	<0.001 [0.64]	89(61.0)_a_	9(5.5)_b_	<0.001 [0.70]
Apprenticeship/vocational training	64(19.9)_a_	33(11.2)_b_		38(14.7)_a_	18(9.8)_a_		31(21.2)_a_	21(12.9)_a_	
High school	41(12.7)_a_	87(29.6)_b_		9(3.5)_a_	48(26.2)_b_		23(15.8)_a_	52(31.9)_b_	
College/university	23(7.1)_a_	130(44.2)_b_		1(0.4)_a_	64(35.0)_b_		3(2.1)_a_	81(49.7)_b_	
Employment status, *n*(%)									
Working	176(54.7)_a_	196(66.7)_b_	0.015 [0.16]	37(14.3)_a_	83(45.4)_b_	<0.001 [0.56]	79(54.2)_a_	111(68.1)_b_	0.12 [0.18]
Student	28(8.4)_a_	16(5.4)_a_		9(3.5)_a_	35(19.1)_b_		8(5.5)_a_	2(1.2)_b_	
Unemployed	25(7.8)_a_	20(6.8)_a_		108(41.9)_a_	19(10.4)_b_		17(11.6)_a_	13(8.0)_a_	
Retired/invalidity pensioner	52(16.0)_a_	44(15.0)_a_		23(8.8)_a_	34(18.6)_b_		17(11.6)_a_	14(7.4)_a_	
Homemaker	16(5.0)_a_	4(1.4)_b_		44(17.1)_a_	7(3.8)_b_		7(4.8)_a_	6(3.7)_a_	
Maternity leave	16(5.0)_a_	6(2.0)_a_		17(6.6)_a_	2(1.1)_b_		14(9.6)_a_	16(9.8)_a_	
Casual worker	10(3.1)_a_	8(2.7)_a_		20(7.8)_a_	1(1.6)_b_		4(2.7)_a_	3(1.8)_a_	
Financial situation, *n*(%)									
Below average	281(87.3)_a_	141(48.0)_b_	<0.001 [0.42]	245(95.0)_a_	124(67.8)_b_	<0.001 [0.38]	99(67.8)_a_	146(31.9)_b_	<0.001 [0.36]
Average	38(11.8)_a_	134(45.6)_b_		13(5.0)_a_	34(18.6)_b_		32(21.9)_a_	50(46.6)_b_	
Above average	3(0.9)_a_	19(6.5)_b_		0(0)_a_	25(13.7)_b_		15(10.3)_a_	74(21.5)_b_	
Marital status, *n*(%)									
Single	55(17.1)_a_	52(17.7)_a_	0.072 [0.12]	48(18.6)_a_	44(24.0)_a_	0.001 [0.21]	26(17.8)_a_	31(19.0)_a_	0.382 [0.12]
Married	105(32.6)_a_	114(38.8)_a_		95(36.8)_a_	84(45.9)_a_		58(39.7)_a_	78(47.9)_a_	
Partnership	79(24.5)_a_	47(16.0)_b_		84(32.6)_a_	27(14.8)_b_		45(30.8)_a_	36(22.1)_a_	
Divorced	42(13.0)_a_	48(16.3)_a_		9(3.5)_a_	11(6.0)_a_		9(6.2)_a_	7(4.3)_a_	
Widowed	41(12.7)_a_	33(11.2)_a_		22(8.5)_a_	17(9.3)_a_		8(5.5)_a_	11(6.7)_a_	
Household composition, *n*(%)									
Single occupancy	51(15.8)_a_	65(22.1)_b_	<0.001 [0.23]	16(6.2)_a_	22(12.0)_b_	<0.001 [0.28]	15(10.3)_a_	20(12.3)_a_	0.698 [0.10]
Married/cohabiting with no dependent children	48(14.9)_a_	85(28.8)_b_		33(12.8)_a_	35(19.1)_a_		39(26.7)_a_	39(23.9)_a_	
Married/cohabiting with dependent children	131(40.7)_a_	82(27.9)_b_		105(40.7)_a_	60(32.8)_a_		53(36.3)_a_	66(40.5)_a_	
Married/cohabiting with dependent children and grandparents	22(6.8)_a_	11(3.7)_a_		52(20.2)_a_	13(7.1)_b_		9(6.2)_a_	7(4.3)_a_	
Single parent family	49(15.2)_a_	28(9.5)_b_		27(10.5)_a_	12(6.6)_a_		10(6.8)_a_	6(3.7)_a_	
Multi-person household with parents	21(6.5)_a_	23(7.8)_a_		25(9.7)_a_	41(22.4)_b_		20(13.7)_a_	25(15.3)_a_	
Number of children, *n*(%)									
Not have children	40(12.4)_a_	75(25.5)_b_	<0.001 [0.27]	21(8.2)_a_	64(35.0)_b_	<0.001 [0.43]	39(26.7)_a_	52(31.9)_a_	0.003 [0.31]
1–2 children	134(41.6)_a_	151(51.4)_b_		94(36.4)_a_	87(47.5)_b_		76(52.1)_a_	89(54.6)_a_	
3–4 children	106(32.9)_a_	57(19.4)_b_		97(37.6)_a_	23(12.6)_b_		19(13.0)_a_	22(13.5)_a_	
5 or more than 5 children	42(13.1)_a_	11(3.7)_b_		46(17.8)_a_	9(4.9)_b_		12(8.2)_a_	0(0)_b_	

*p*[ES], *p*-value with effect size index. For age Hedges’s g effect size index was calculated, for all other variables, Cramer’s V was calculated to measure the strength of association. Column percentages are reported by comparing column proportions. Each subscript letter denotes a subset of ethnicity categories whose column proportions do not differ significantly from each other at the 0.05 level.

### 3.2 Attendance at any kind of screening tests among Roma and non-Roma

**Figure 2:**
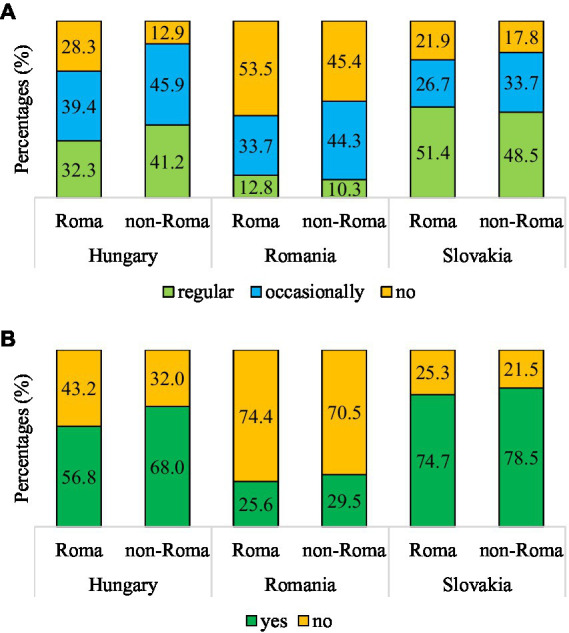
Association between ethnicity and attendance at any kind of screening tests **(A)**, and attendance at any kind of screening tests in the year before the survey **(B)** in Hungary, Romania and Slovakia. **(A)** Hungary: *χ*^2^(2,*N* = 616) = 22.077, *p* < 0.001, *V* = 0.19, Romania: *χ*^2^(2,*N* = 441) = 5.063, *p* = 0.080, *V* = 0.11, Slovakia: *χ*^2^(2,*N* = 309) = 2.046, *p* = 0.360, *V* = 0.08. **(B)** Hungary: *χ*^2^(1,*N* = 616) = 8.190, *p* = 0.005, Φ = 0.19, Romania: *χ*^2^(1,*N* = 441) = 0.833, *p* = 0.386, Φ = 0.04, Slovakia: *χ*^2^(1,*N* = 309) = 0.645, *p* = 0.501, Φ = 0.05.

Analysing the ASBS, it was found that the majority of the Romanian population, regardless of ethnicity, did not attend at any kind of screening tests (72.8%, *n* = 321). In Hungary, this rate was 37.8% (*n* = 233), and in Slovakia, it was 23.3% (*n* = 72). There was a significant association between ethnicity and ASBS in Hungary (*χ*^2^(1,*N* = 616) = 8.190, *p* = 0.005, Ф = 0.12). Specifically, a higher proportion of Roma women (43.2%, *n* = 139) did not attend at any kind of screening tests compared to non-Roma women (32%, *n* = 94) (Figure 2).

Furthermore, we examined the main reasons for not attending at any kind of screening tests. In Hungary and Slovakia, irrespective of ethnicity, the primary reason reported was ‘disliking visits to the doctor.’ This was also the main reason among non-Roma women in Romania. However, among Roma women in Romania, the main reason for not attending at any kind of screening tests was the lack of health insurance.

### 3.3 Factors influencing attendance at any kind of screening tests among Roma and non-Roma women

In our study, we examined several factors that were hypothesized to influence ASBS, including having health insurance, subjective health status, having chronic disease(s), and health behaviours such as smoking and physical activity.

#### 3.3.1 Having health insurance

An association was found between ASBS and having health insurance among Roma women in Romania. Roma women without health insurance had a lower rate of ASBS compared to those with health insurance. It is important to note that there is a higher proportion of women in Romania who do not have health insurance, regardless of ethnicity. Furthermore, a significant association was observed between having health insurance and ASBS in Hungary, although this association was not significant when considering ethnicity independently (Table 2).

**Table 2:** The associations between attendance at any kind of screening tests in the year before the survey (ASBS) and health insurance, health status, chronic diseases, smoking and physical activity in Roma and non-Roma women by country.

		Health insurance		*χ* ^2^	*p* [Φ]	Health status		*χ* ^2^	*p* [Φ]	Chronic diseases		*χ* ^2^	*p* [Φ]	Smoking		*χ* ^2^	*p* [Φ]	Physical activity		*χ* ^2^	*p* [Φ]
		Yes	No			Healthy	Ill			Yes	No			Yes	No			Yes	No		
Hungary Roma																					
ASBS	Yes	58.4 (167)_a_	44.4 (16)_a_	2.535	0.152 [0.09]	54.5 (90)_a_	59.2 (93)_a_	0.721	0.431 [0.15]	62.0 (129)_a_	47.4 (54)_b_	6.443	0.013 [0.14]	51.6 (99)_a_	64.6 (84)_b_	5.383	0.022 [0.13]	61.7 (66)_a_	54.4 (117)_a_	1.536	0.234 [0.07]
	No	41.6 (119)_a_	55.6 (20)_a_			45.5_a_ (75)_a_	40.8 (64)_a_			38.0 (79)_a_	52.6 (60)_b_			48.4 (93)_a_	35.4 (46)_b_			38.3 (41)_a_	45.6 (98)_a_		
Hungary non-Roma																					
ASBS	Yes	69.1 (183)_a_	58.6 (17)_a_	1.309	0.295 [0.07]	69.2 (148)_a_	65.0 (52)_a_	0.463	0.574 [0.04]	70.4 (119)_a_	64.8 (81)_a_	1.041	0.315 [0.06]	62.0 (44)_a_	70.0 (156)_a_	1.578	0.243 [0.07]	68.9 (84)_a_	67.4 (116)_a_	0.065	0.899 [0.02]
	No	30.9 (82)_a_	41.4 (12)_a_			30.8 (66)_a_	35.0 (28)_a_			29.6 (50)_a_	35.2 (44)_a_			38.0 (27)_a_	30.0 (67)_a_			31.1 (38)_a_	32.6 (56)_a_		
Romania Roma																					
ASBS	Yes	34.0 (34)_a_	20.3 (32)_b_	6.079	0.019 [0.15]	24.0 (23)_a_	26.5 (43)_a_	0.212	0.768 [0.03]	31.9 (53)_a_	14.1 (13)_b_	9.849	0.002 [0.20]	24.8 (39)_a_	26.7 (27)_a_	0.116	0.771 [0.02]	24.0 (18)_a_	26.2 (8)_a_	0.139	0.755 [0.02]
	No	66.0 (66)_a_	79.7 (126)_b_			76.0 (73)_a_	73.5 (119)_a_			68.1 (113)_a_	85.9 (79)_b_			75.2 (118)_a_	73.3 (74)_a_			76.0 (57)_a_	73.8 (135)_a_		
Romania non-Roma																					
ASBS	Yes	31.6 (50)_a_	16.0 (4)_a_	2.540	0.156 [0.12]	32.4 (34)_a_	25.6 (20)_a_	0.977	0.413 [0.07]	33.3 (35)_a_	24.4 (19)_a_	1.733	0.195 [0.10]	26.1 (12)_a_	30.7 (42)_a_	0.346	0.709 [0.04]	41.7 (25)_a_	58.3 (35)_b_	6.344	0.016 [0.19]
	No	68.4 (108)_a_	84.0 (21)_a_			67.6 (71)_a_	74.4 (58)_a_			66.7 (70)_a_	75.6 (59)_a_			73.9 (34)_a_	69.3 (95)_a_			58.3 (35)_a_	76.4 (94)_b_		
Slovakia Roma																					
ASBS	Yes	80.2 (105)_a_	26.7 (4)_b_	20.350	<0.001 [0.37]	76.4 (84)_a_	69.4 (25)_a_	0.686	0.508 [0.07]	81.2 (69)_a_	65.6 (40)_b_	4.570	0.036 [0.08]	54.8 (23)_a_	82.7 (86)_b_	12.336	0.001 [0.29]	77.8 (49)_a_	72.3 (60)_a_	0.570	0.565 [0.06]
	No	19.8 (26)_a_	73.3 (11)_b_			23.6 (26)_a_	30.6 (11)_a_			18.8 (16)_a_	34.4 (21)_b_			45.2 (19)_a_	17.3 (18)_b_			22.2 (14)_a_	27.7 (23)_a_		
Slovakia non-Roma																					
ASBS	Yes	79.7 (122)_a_	60.0 (6)_a_	2.169	0.224 [0.12]	79.0 (98)_a_	76.9 (30)_a_	0.078	0.824 [0.02]	84.7 (72)_a_	71.8 (56)_b_	4.021	0.056 [0.16]	69.0_a_ (20)	80.6_a_ (108)	1.913	0.221 [0.11]	82.7 (62)_a_	75.0 (66)_a_	1.411	0.256 [0.09]
	No	20.3 (31)_a_	40.0 (4)_a_			21.0 (26)_a_	23.1 (9)_a_			15.3 (13)_a_	28.2 (22)_b_			31.0_a_ (9)	19.4_a_ (26)			17.3 (13)_a_	25.0 (22)_a_		

Data presented in %(*n*), *χ*^2^: chi-square test of independence, *p*: *p*-value, Fisher’s exact test, Φ: Phi effect size index. Column percentages are reported by comparing column proportions. Each subscript letter denotes a subset of categories (health insurance, health status, chronic diseases, smoking, physical activity) whose column proportions do not differ significantly from each other at the 0.05 level.

#### 3.3.2 Health status and having chronic disease(s)

Subjectively reported health status showed a non-significant association with ASBS. However, having chronic disease(s) was significantly associated with ASBS among Roma women in all three countries. Among Roma women, a higher proportion of those with chronic disease(s) had undergone at any kind of screening tests compared to those without chronic disease(s), even if they did not have health insurance (Table 2).

#### 3.3.3 Health behaviours

A significant association was found between smoking and ASBS in Hungary and Slovakia among Roma women. A higher proportion of non-smoking or former smoking Roma women had undergone any kind of screening tests in the year before the survey compared to current smokers. However, in Romania, there was no significant association between smoking and ASBS for both Roma and non-Roma women (Table 2).

Examining an active lifestyle, we compared women who engaged in regular exercise (daily or several times a week) to those who did not. Among non-Roma women in Romania, there was a significant association between an active lifestyle and ASBS. A higher proportion of active women had undergone any kind of screening tests in the year before the survey (41.7%, *n* = 25) compared to their non-active counterparts (23.6%, *n* = 29). However, in Hungary and Slovakia, the association between an active lifestyle and ASBS was not significant (Table 2).

For socio-economic characteristics and examined factors between Roma and non-Roma regardless of the countries, see Supplementary File S2.

### 3.4 Predictors of attendance at any kind of screening tests in Roma and non-Roma women by country

Multiple binary logistic regression models were constructed separately for each country and ethnicity to examine the effects of health insurance, health status, chronic disease(s), and health behaviours on the likelihood of attending at any kind of screening tests (refer to Table 3).

**Table 3:** Predictors of attendance at any kind of screening tests in the year before the survey in Roma and non-Roma women by country.

		Hungary		Romania		Slovakia	
		Roma	non-Roma	Roma	non-Roma	Roma	non-Roma
		aOR (95%CI)		aOR (95%CI)		aOR (95%CI)	
Health insurance	Yes	ref	ref	ref	ref	ref	ref
	No	0.60 [0.29;1.22]	0.64 [0.29;1.42]	**0.50 [0.27;0.91]**	0.49 [0.15;1.56]	**0.09 [0.02;0.36]**	0.36 [0.09;1.43]
Chronic diseases	Yes	**1.71 [1.01;2.90]**	1.41 [0.83;2.39]	**2.87 [1.44;5.72]**	1.76 [0.86;3.61]	1.73 [0.72;4.14]	**2.52 [1.12;5.66]**
	No	ref	ref	ref	ref	ref	ref
Health status	Feel healthy	ref	ref	ref	ref	ref	ref
	Feel ill	1.07 [0.64;1.79]	0.78 [0.43;1.41]	0.93 [0.49;1.74]	0.62 [0.31;1.27]	1.02 [0.36;2.89]	0.82 [0.33;2.01]
Smoking	Yes	**0.57 [0.35;0.91]**	0.74 [0.42;1.31]	1.07 [0.58;1.96]	0.96 [0.43;2.11]	**0.25 [0.11;0.61]**	0.44 [0.17;1.14]
	No	ref	ref	ref	ref	ref	ref
Physical activity	Yes	ref	ref	ref	ref	ref	ref
	No	0.76 [0.47;1.23]	0.96 [0.58;1.59]	1.10 [0.57;2.13]	**0.48 [0.25;0.95]**	0.70 [0.29;1.68]	0.69 [0.31;1.55]

aOR [95%CI]: adjusted odds ratio with 95% confidence interval, ref: reference category. Bold: significant predictors, *p* < 0.05.

In Hungary, the model was significant only for Roma women (*χ*^2^(5) = 15.213, *p* = 0.009). Having chronic disease(s) was associated with an increased likelihood of attending at any kind of screening tests, while smoking decreased the odds of attendance by 43.2%.

In Romania, both Roma and non-Roma women showed significant associations with ASBS (Roma: *χ*^2^(5) = 16.216, *p* = 0.006; non-Roma: *χ*^2^(5) = 11.153, *p* = 0.048). Among Roma women, not having health insurance reduced the odds of attendance by 50.4% and having chronic disease(s) increased the likelihood of attendance. Among non-Roma women, physical inactivity decreased the odds of attendance by 51.6%.

In Slovakia, among Roma women, not having health insurance and smoking significantly decreased the odds of attending at any kind of screening tests by 90.6 and 74.8%, respectively (*χ*^2^(5) = 30.224, *p* < 0.001). Among non-Roma women, having chronic disease(s) was associated with an increased likelihood of attending at any kind of screening tests, although the association was not statistically significant (*χ*^2^(5) = 10.068, *p* = 0.073).

## 4 Discussion

### 4.1 Having health insurance and access to healthcare

Our study revealed that the proportion of women with health insurance is relatively high in Hungary and Slovakia, while less than half of Roma women in Romania have health insurance. We found a significant association between health insurance and attendance at any kind of screening tests among Roma women in Romania. This suggests that lacking health insurance decreases the likelihood of Roma women attending at any kind of screening tests and highlights the issue of unmet health needs for this population. In Slovakia, where the majority of Roma have health insurance, those without insurance are being excluded from the healthcare system and therefore not accessing screening tests. These findings align with reports from the European Union Agency for Fundamental Rights and previous studies that emphasize the challenges faced by the Roma population in accessing healthcare (11, 26).

### 4.2 Chronic disease(s) and access to healthcare

In Bosnia and Herzegovina, a survey revealed a high proportion of health insurance coverage among Roma women, similar to our findings in Hungary and Slovakia. However, despite having health insurance, a significant number of Roma individuals with chronic disease(s) were not under medical supervision, leading to a lack of regular screening tests. In contrast, our study in Hungary showed that Roma women with chronic disease(s) were more likely to attend at any kind of screening tests, indicating a certain level of awareness regarding their health conditions. This finding was not observed in Slovakia, where non-Roma women with chronic disease(s) were more likely to attend at any kind of screening tests. It is interesting to note that in Hungary and Slovakia, smoking among Roma individuals was associated with a lower likelihood of attending screening tests, suggesting that smoking may serve as a coping mechanism to avoid confronting potential health issues (30).

### 4.3 Healthcare barriers and socio-economic factors

Our study highlighted the challenges arising from low socio-economic status in Romania, including poverty, lower education levels, lower health literacy, and communication difficulties. These factors contribute to limited access to healthcare services (17–23). In terms of educational status, income, and employment, the Romanian results were the least favourable among the three countries. These findings underscore the need to address socio-economic disparities to improve healthcare access and screening tests participation.

### 4.4 Information about any kind of screening tests

The lack of information about any kind of screening tests emerged as a common barrier in all three countries, with a notable proportion of both Roma and non-Roma residents reporting insufficient knowledge about any kind of screening tests. Addressing this issue requires efforts to improve awareness and understanding of screening procedures and benefits among the Roma population. Additionally, reducing the shame associated with the screening process could positively influence participation rates (40). The existence of organized, invitation-based screening tests for breast and cervical cancer is promising, but challenges related to accurate address-based registration and delivery of invitations to residents in Roma communities need to be addressed (35, 40).

### 4.5 Promising initiatives

Hungary’s successful initiative of integrating cervical screening with the support provided by health visitors, who have established trust with mothers in segregated areas, demonstrates the potential of targeted interventions to increase screening rates (40). In Romania and Slovakia, health mediation programs have been implemented to improve Roma health outcomes by enhancing access to healthcare and participation in public health interventions. These programs have proven effective in promoting the inclusion of the Roma minority (27, 33–35). Furthermore, increasing knowledge and cultural sensitivity among healthcare workers, along with promoting the participation of Roma individuals in health professions, could contribute to better communication and understanding between healthcare providers and Roma patients (24, 25).

### 4.6 Strengths and limitations

The strengths of our research lie in the significant number of Roma participants and the inclusion of a comparable sample of non-Roma respondents from the same areas. This is particularly noteworthy considering the challenge is associated with reaching and involving the Roma population in scientific research. Despite the difficulties posed by the COVID-19 pandemic and the unique characteristics of the Roma population, we were able to achieve the desired number of participants from all three countries. However, limitations include the inability to calculate response rates among Roma women due to the difficulty in determining the total female population of this group. Additionally, the long duration of data collection due to the pandemic and the challenges of accessing the special Roma population should be acknowledged.

## 5 Conclusion

**Figure 3:**
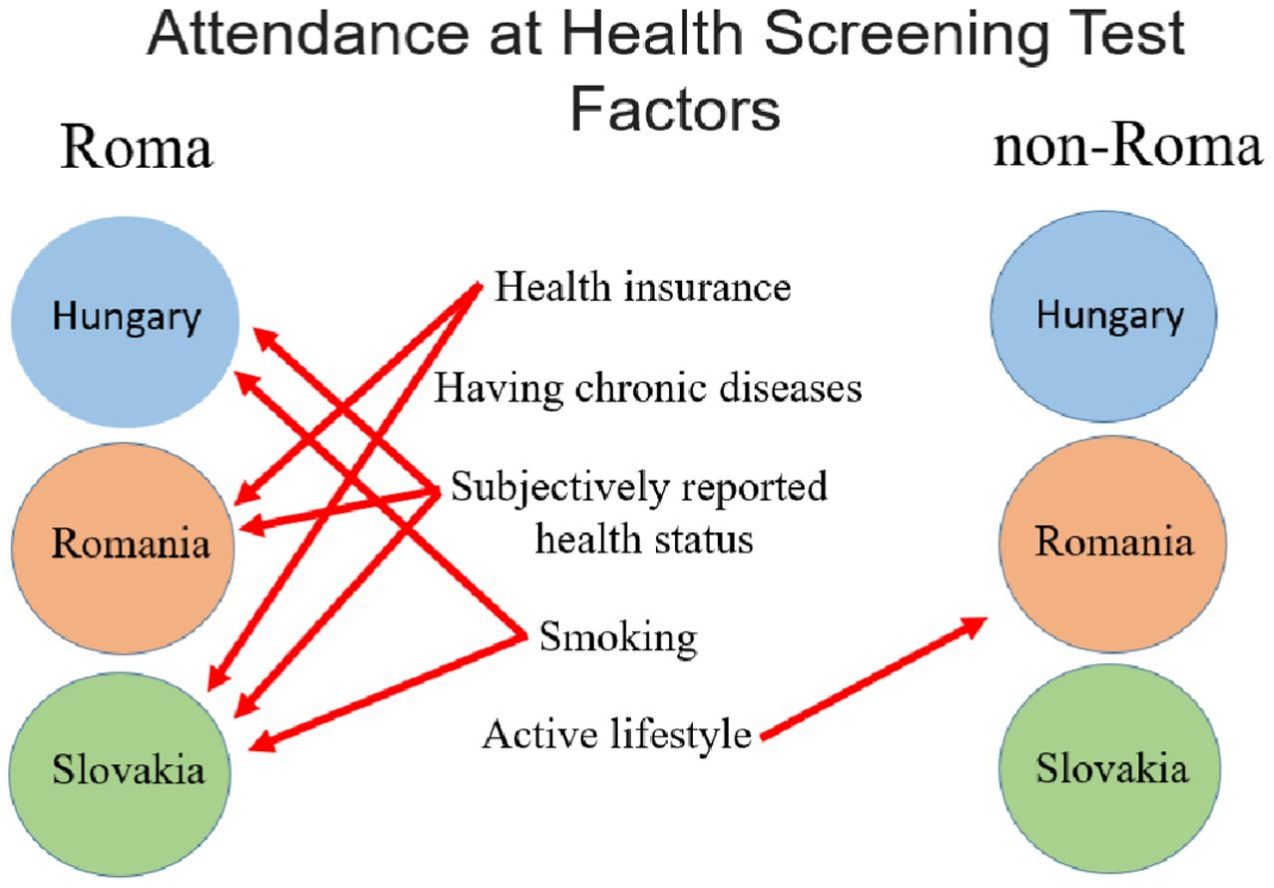
Factors which influence (*p* < 0.005) the attendance at health screening tests.

Our study revealed both similarities and differences in attendance at any kind of screening tests among Roma women in the three countries (Figure 3).

The lack of health insurance was identified as a crucial factor influencing attendance, highlighting the need to ensure insurance coverage for Roma individuals. The COVID-19 pandemic has further exacerbated the challenges faced by individuals with insurance, underscoring the importance of new public health programs to encourage screening participation post-pandemic. Efforts should also be directed towards providing targeted information to Roma communities to increase awareness and participation in screening tests programs. It is essential to enhance healthcare providers’ knowledge, sensitivity, and positive attitudes towards the Roma population to improve communication and reduce stigma and discrimination.

The findings suggest that it would be important to change the health perceptions of the Roma population in all three countries, as attendance at any kind of screening tests was significantly higher among people with chronic diseases even if they did not have health insurance. According to Roma’s perceptions of illness, visible and perceptible symptoms are the presence of an illness for which a doctor should be consulted, so according to this belief, if they do not experience symptoms, they cannot be ill. This attitude discourages the use of screening tests, so education about the effectiveness of these tests is essential.

Our results show that health behaviours, such as not smoking, increase the attendance rate of any kind of screening tests among Roma. So, living a health-conscious lifestyle has a beneficial effect on several areas of one’s life, including attendance at screening tests. For this reason, in addition to providing information on screening tests for Roma, we believe it is important to create a complex health promotion programme that covers a range of topics such as healthy eating, exercise and harmful addictions.

Further research with larger sample sizes in other countries with Hungarian-speaking Roma and non-Roma minorities is warranted to gain a comprehensive understanding of the health behaviour and needs of these populations. The findings of our gap-filling study shed light on persisting issues while providing valuable insights into the hidden minority of Hungarian-speaking Roma.

## Data availability statement

The raw data supporting the conclusions of this article will be made available by the authors, without undue reservation.

## Ethics statement

The studies involving humans were approved by Medical Research Council, Committee on Scientific Research Ethics, Hungary Number: ETT TUKEB IV/3495-4/ 2021/EKU. The studies were conducted in accordance with the local legislation and institutional requirements. Written informed consent for participation was not required from the participants or the participants’ legal guardians/next of kin in accordance with the national legislation and institutional requirements.

## Author contributions

NM: Conceptualization, Writing – original draft. JT: Data curation, Formal analysis, Methodology, Visualization, Writing – original draft. ZU: Supervision, Writing – review & editing. HF: Conceptualization, Supervision, Writing – review & editing.
