# Supplementary figures and images for: Screening attendance disparities among Hungarian-speaking Roma and non-Roma women in central and eastern European countries
Source: Front Public Health. 2023 Dec 22;11:1292598. doi: 10.3389/fpubh.2023.1292598 (PMC10771837; doi:10.3389/fpubh.2023.1292598)

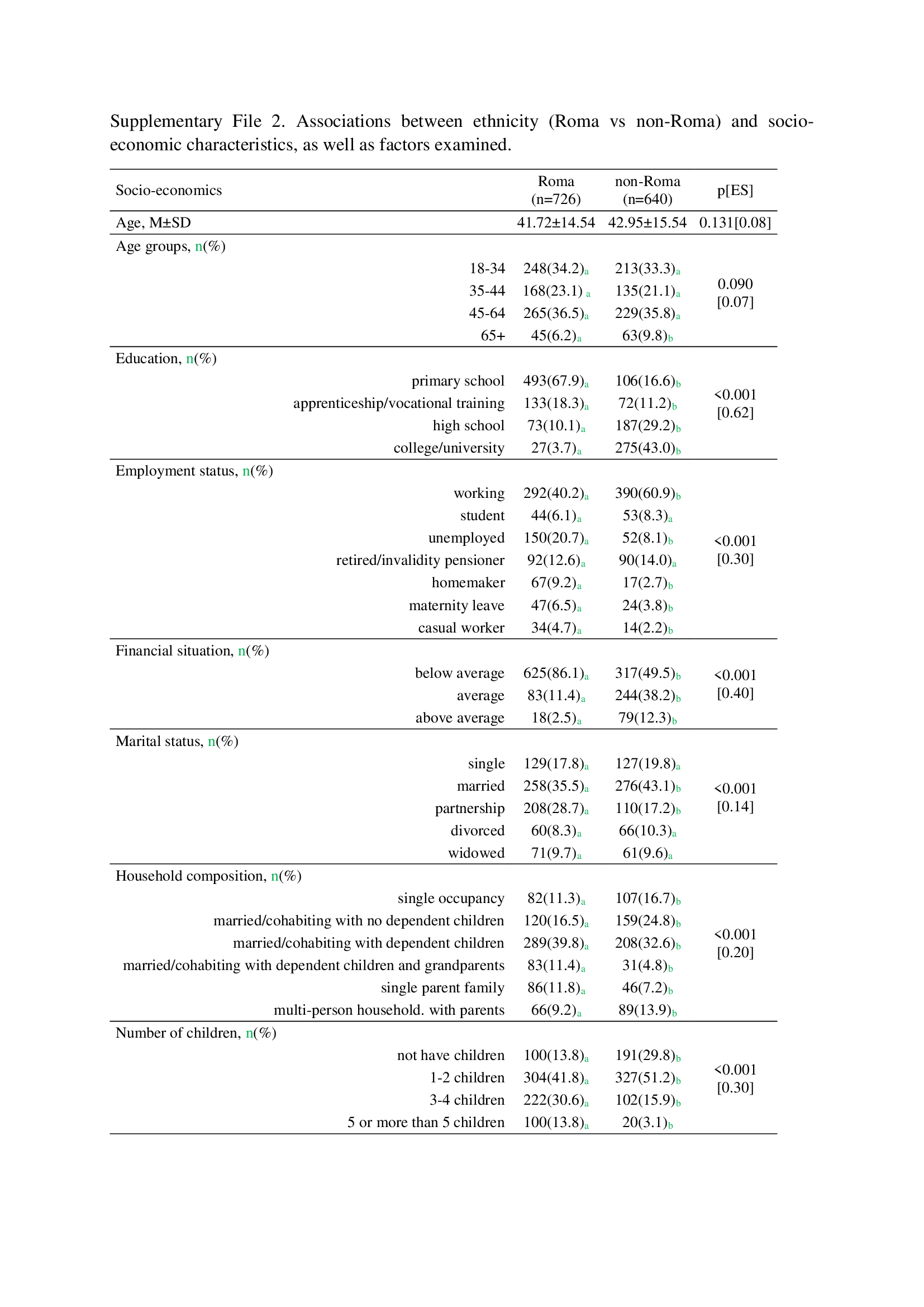

Supplement: Supplementary file 2 [file Image_2.TIFF]
